# Supplementary material for: CRISPR-mediated Transfection of Brugia malayi
Source: PLoS Negl Trop Dis. 2020 Aug 31;14(8):e0008627. doi: 10.1371/journal.pntd.0008627 (PMC7485969; doi:10.1371/journal.pntd.0008627)
Supplement: Attachment — Submitted filename: Liu et al rev cover.docx [file pntd.0008627.s002.docx]

June 12, 2020

Sabine Specht
Associate Editor
Timothy Geary
Deputy Editor
PLOS Neglected Tropical Diseases

Dear Drs. Specht and Geary;

Thank you for your e mail of June 2, 2020 regarding our manuscript entitled “CRISPR-mediated Transfection of *Brugia malayi*”. Accompanying this letter, please find a revised version of the manuscript (track changes enabled and clean) in which we have addressed all the reviewer’s concerns regarding the original submission. In the paragraphs below, I will take the opportunity of summarizing the reviewers’ comments and our response to them.

**Reviewer 2:**

I am going to take the reviewers out of order, because reviewer 2 stated “This is definitely the most important paper in filarial biology that I have read this year.  It is an excellent follow-up to their recent paper on successful transfection of *Brugia malayi*”, and had minor corrections, which the reviewer included as a track changes marked up version of the manuscript. We used the reviewer’s marked up document to prepare our revision and started by accepting the reviewer’s changes. Thus, the revision contains this reviewer’s suggested minor modifications, but they do not show up as tracked changes.

**Reviewer 1:**

1. Reviewer 1’s major concern was that we did not include a control showing the amount of secreted luciferase in medium from microfilariae collected from animals infected with untransfected parasites. This was an oversight on our part, and we now include the requested control, both in the text and in a modified version of Figure 3.

2. The reviewer also felt that the manuscript would be much improved if we included a discussion of the larger picture of the recent applications of CRISPR in other parasitic nematodes and trematodes. We have done so, including a paragraph in the Introduction that discusses the advances in CRISPR in other parasitic worms.

3. The reviewer felt that we should include the sizes of the arms included in the plasmid to direct the homologous recombination. The reviewer suggested including this in several places in the manuscript. We have done so, adding this information in all places suggested by the reviewer.

4. The reviewer suggested including a mention of the concept of “genomic safe harbors” as part of the rationale for how we chose our insertion sites. We have done so.

5. The reviewer pointed out that the size of the arms might have an effect on the efficiency of the process and suggested we put this in context of what has been found in other parasitic worms. We have now included a paragraph in the discussion that does as the reviewer suggests.

6. The reviewer suggested we re-label Figure 2 to make the contents of the wells easier to interpret. We have done so.

7. The reviewer pointed out that the *piggyBac* system might result in multiple integrations in the genome confounding any analysis of gene expression from the integrated transgenes. We now include this point in the revision.

8. The reviewer suggested that we might mention the potential of using a modified florescent cell sorter to collect transgenic worms. We struggled whether to include this or not and decided not to include it. The reason for this is that we have been aware of this and have been trying to use the published *C. elegans* protocol to sort transgenic microfilariae. So far, it’s been an expensive disaster – the microfilariae are quite motile, which is interfering with the collection (they seem to be getting sprayed all over the inside of the hood enclosing the sorter) and the ones that do get collected are in bad shape and do not survive for very long. So, we have tried this out, and so far it has not worked at all. But this does not mean that if the process was optimized, it might not actually work. We hate to mention this possibility without talking about our experience, but if we mention our experience we may discourage others from trying this out and succeeding. So, we have decided in the end that it is for the best not to mention it here.

9. The reviewer raised several minor points of grammar and language. We have made all the corrections noted by the reviewer in the revision.

We hope that the changes we have made in the revision will render this manuscript acceptable for publication in *PLoS Neglected Tropical Diseases*. Thank you once again for considering this manuscript.

Sincerely yours,

Thomas R. Unnasch, PhD, FASTMH

Corresponding author
